# Ethnopharmacological Survey, Phytochemical Screening and Antimicrobial Activities of Medicinal Plants Used in the Treatment of Diarrhea in Southeastern Gabon

**DOI:** 10.3390/plants12203629

**Published:** 2023-10-20

**Authors:** Jean Fabrice Yala, Rolande Mabika Mabika, Davy U. Ikabanga, Franck Mounioko, Prince Rodrigue Mokouapamba, Alexis Nicaise Lepengue, Alain Souza

**Affiliations:** 1 Bacteriology Laboratory, Unity for Research Medical Analysis of the Interdisciplinary, Centre of Medical Research of Franceville (CIRMF), Franceville BP 769, Gabon; rolandemabika@gmail.com; 2 Laboratory of Molecular and Cellular Biology, Bacteriology-Immunology Team, Agrobiology Research Unit, University of Science and Technology of Masuku (USTM), Franceville BP 067, Gabon; fmounioko@yahoo.fr (F.M.); mokouapambap@yahoo.fr (P.R.M.); souzapg@yahoo.fr (A.S.); 3 Laboratory of Plant Physiology, Phytopathology and Plant Breeding, Agrobiology Research Unit, University of Science and Technology of Masuku (USTM), Franceville BP 067, Gabon; davyulrich2009@yahoo.fr (D.U.I.); lepengue_nicaise@yahoo.fr (A.N.L.); 4 Unity for Research Health Ecology of the Interdisciplinary, Centre of Medical Research of Franceville (CIRMF), Franceville BP 769, Gabon; 5 Laboratory of Animal Physiology and Pharmacology, Agrobiology Research Unit, Masuku University of Science and Technology (USTM), Franceville BP 067, Gabon

**Keywords:** ethnopharmacological efficacy, antimicrobial activity, phytochemical screening, anti-diarrheal plants

## Abstract

Diarrhea is a condition that particularly affects children under five the age of years old in developing countries. The aim of this study was to evaluate the efficacy of medicinal extracts plants used in treatment and to characterize their inhibitory capacity in the growth of bacteria isolates in childhood diarrhea in the southeast region of Gabon. An ethnobotanical survey on the plants used in the treatment of diarrhea in southeastern Gabon was carried out and a phytochemical screening of the collected plants was performed. The antimicrobial activity of aqueous extracts was evaluated; 33 plant species were identified, representing 28 genera and 19 families. Bark (39.5%) was the most commonly used plant part, followed by powder formulations (28.9%). The preferred method of preparation and chewing (44.7%), together with drinking (36.8%), were the most prevalent modes of administration. Phytochemical screening showed a plethora of secondary metabolites (terpenoids, alkaloids and phenolic compounds), and a marked level of anti-diarrheal activity was found with *Sida acuta* and *Santiria. ebo* against *Shigella* spp. (16.22 ± 0.7 mm and 14.05 ± 1.4 mm) and *Yersinia pestis* (16.35 ± 0.5 mm and 15.51 ± 0.5 mm). The high diversity of secondary plant metabolites and their inhibitory ability against enteric pathogens would justify their use at the local level to treat diarrheal infections.

## 1. Introduction

Infant-related diseases remain a major public health problem worldwide. According to the World Health Organization (WHO), pneumonia and diarrhea are among the main causes of death in infants, accounting for 29% of all deaths in children under five years old [1,2]. Diarrheal diseases are a threatening health issue with an increasing magnitude, prevalence and incidence [1,2,3,4]. Approximately, 760,000 infantile-related deaths are annually attributed to diarrheal diseases. Africa and Southeast Asia have the highest rates of infant mortality with diarrhea present in 80% of cases [3]. Due to their pathophysiological manifestations, an additional estimate of 1.3 billion diarrheal episodes occur each year worldwide [4]. Although the existence of non-infectious diarrhea is widespread, bacterial pathogens are one of the major causes of infectious diarrheal diseases, making infectious diarrhea the most common form of the disease [1,2]. When treating infectious forms of the disease, the misuse of anti-diarrheal drugs has led to the development of multi-resistant bacteria [5]. As a consequence, the use of certain antibiotics is prohibited because of elevated rates of therapeutic failure. Since 1978, the United Nations Children’s Fund (UNICEF) and the WHO have advocated oral rehydration therapy (ORT) as the main treatment modality for diarrheal symptoms and dehydration, which is the main complication [6]. Several studies have reported an increasing use of traditional medicine by populations in developing countries due to multiple aspects, including the high expensive cost of pharmaceutical drugs, the absence of adequate health centers and the easy access to medicinal plants, [1,7,8]. The continuous use of medicinal plants could offer promising alternatives to ineffective antibiotics, coupled with the discovery of new potent bioactive molecules and components. These would facilitate the development and acceptation of improved traditional medicines (IMT) in the short term.

The floristic diversity in the dense rainforest of Gabon and the preferential use of medicinal plants by the Gabonese population offer promising research perspectives. Indeed, a great diversity of plants is used for their anti-diarrheal properties. However, no study has yet been conducted on the in vitro antibacterial activities of these plants on the bacteria responsible for diarrheal diseases. Therefore, the phytochemical screening of plant species in Gabon was necessary to determine their antibacterial activity and potency.

The purpose of this study was to identify plant species with potential anti-diarrheal properties, as well as acquire additional knowledge on the flora of Gabon. The main objectives were as follows: (i) to inventory the medicinal plants used in the treatment of diarrhea in southeast Gabon; (ii) to carry out phytochemical screening of the identified medicinal plants; and (iii) to assess their antibacterial activity against diarrheal pathogens.

## 2. Results

### 2.1. Ethnopharmacological Survey

#### 2.1.1. Informants

During the study period of 8 weeks, 108 adults were interviewed, including 48 men (44.44%) and 60 women (55.56%). Moreover, the results of this survey underline that 55.56% (60/108) of participants had adequate knowledge of the medicinal plants used in the treatment of diarrheal infections, with 32.41% of women against 23.25% of men (Table 1).

#### 2.1.2. Diversity and Specific Abundance of the Anti-Diarrheal Plants

From the ethnobotanical survey, 38 plants were identified that are used in the treatment of diarrheal infections. Of these plants, 33 were further categorized and included 28 genera and 19 families. The analysis of the results in Table 2 shows a specific diversity of families of plants used against diarrheal agents in southeast Gabon. *Burseraceae* (16.2%) and *Asteraceae* (10.8%) are the most represented families, followed by *Annonaceae* and *Verbenaceae* (8.1% each), and *Rubiaceae* and *Fabaceae* (5.4% each). The most used genera in the treatment of diarrheal infections in southeastern Gabon are *Dacryodes* (7.9%), *Annona* (5.3%), *Lippia* (5.3%), and *Santiria* (5.3%).

#### 2.1.3. Plant Extracts Used for the Treatment of Diarrhea

Information on the selection of used plant parts, the methods of preparation and their administration were reported and are given in Table 3.

Table 3 shows that bark (39.5%) was the part of the plants most frequently used for the preparation of anti-diarrheal drugs; the rest are in decreasing order as follows: leaves (34.2%); roots (7.9%); fruits (7.9%); core of the trunk (2.6%); core of the plant (2.6%); twig leaf (2.6%); and seeds (2.6%). The preferred methods of preparation were bark powder (28.9%) and decoction (26.3%), while maceration (13.2%) and raw plant part intake (5.3%) were moderately used as the common modes of administration by the study population. Other preparation methods (2.6%) were seldom used. Chewing (44.7%), drinking (36.8%) and purging (5.3%) were also used as methods of administration (Table 3).

### 2.2. Phytochemical Screening

Out of the 33 plants identified in the ethnobotanical survey, 20 plants were selected and subjected to phytochemical screening. Table 4 presents the results of the phytochemical screening of the extracts from the 20 selected plants.

Analysis of the phytochemical screening of the aqueous extracts of 20 anti-diarrheal plants revealed the richness in secondary metabolites, which depends on the plant or the parts of the plant that is used. Indeed, the aqueous extract of the leaves of *Annona senegalensis* was rich in terpene/sterols, reducing sugars and polyphenols while containing tannins. On the other hand, saponosides, flavonoids, alkaloids, anthracene compounds and cardiotonic heterosides were absent in this extract. The aqueous extract of the leaves of *Ageratum conyzoides* was rich only in terpenes/sterols and contained a low level of polyphenols, tannins, alkaloids and cardiotonic heterosides, but secondary metabolites were absent. The extract of the bark of *Anonidium mannii* contained polyphenols, terpenes/sterols and a low level of tannins in abundance, but saponosides, flavonoids, alkaloids, anthracene compounds and cardiotonic heterosides all could not be traced as they were absent. The aqueous extracts of the leaves of *Bidens pilosa* and *Cissus leonardii* had an abundance of polyphenols, tannins, alkaloids and terpenes/sterols. However, the flavonoids and reducing sugars that were abundant in the leaves of *Bidens pilosa* were only found in low amounts in the leaves of *Cissus leonardii*. Furthermore, the anthracene compounds weakly present in the extract of the leaves of *Bidens pilosa* were very abundant in that of *Cissus leonardii.* These two extracts did not contain saponosides. The bark extracts of *Pachyylobus buettneri* and *Pachyylobus camerunenis* were rich in saponosides, polyphenols, tannins, alkaloids, terpenes/sterols, anthracene compounds and cardiotonic heterosides. They were poor in flavonoids. However, the reducing sugars abundant in the extract of *Pachyylobus camerunensis* were only found in low levels in that of *Pachyylobus buettneri*. The aqueous extracts of the bark of *Santiria ebo* and of *Santiria trimera* abundantly contained polyphenols, tannins, terpenes/sterols, anthracene compounds, mainly flavonoids and cardiotonic heterosides.

A comparison of the phytochemical composition of extracts from the pulp of *Alchornea cordifolia* fruit and leaves showed that extracts were rich in polyphenols, tannins, alkaloids, terpenes/sterols, anthracene compounds and reducing sugars, but only a weak presence of cardiotonic heterosides was recorded. On the other hand, saponosides and flavonoids were abundant in the pulp of the fruit but were absent and in trace amounts in the leaves, respectively. These secondary metabolites were very abundant in extracts of the roots of *Carica papaya* and the leaves of *Psidium guineense*, with the exception of tannins and reducing sugars. Phytochemical screening revealed that the aqueous extract of the leaves of *Geophila renaris* mainly contained saponosides and reducing sugars and, in the majority, polyphenols. In addition, the extract of seeds of *Persea americana* and the bark of *Pauridiantha callicarpoides* had the same qualitative composition as secondary metabolites, although the flavonoids that were very weakly present in the extract of *Pauridiantha callicarpoides* were absent in that of *Persea americana*. The extract from the leaves of *Tetracera alnifolia* was rich in polyphenols, tannins, terpenes and reducing sugars. It contained a low level of saponosides and cardiotonic heterosides but did not contain other metabolites. While the bark extract of *Scottellia klaineana* abundantly contained polyphenols, terpenes/sterols, cardiotonic heterosides and reducing sugars, there were weak levels of anthracene compounds and no presence of saponosides, tannins, flavonoids and alkaloids. Finally, the aqueous extract of the bark of *Pycnantus angolensis* did not contain tannins, only low levels of saponosides, flavonoids and reducing sugars.

### 2.3. Antimicrobial Activities of Plant Extracts Studied

The antibacterial activity of the aqueous extracts of the plants was evaluated by measuring the inhibitory power or antibacterial activity of 10 plants at the same concentration on the bacterial strains (pathogens and opportunistic pathogens). The results obtained are listed in Table 5, revealing that the extracts from have an inhibitory effect on the growth of the tested microorganisms. However, this antibacterial activity varies depending on the bacterial type and the plant extract.

#### 2.3.1. Opportunistic Pathogens

Overall, the results show that 7 out of 10 plants had antibacterial activity on all the opportunistic pathogens studied (5 strains). However, the extract of the leaves of Alchornea cordifolia was active only on Escherichia vulneris and Serratia odorifera, while those of Santiria trimera, Carica papaya and Cissus leonardii showed no antibacterial activity on the five strains. The results of the activity of gentamicin on the five bacterial strains (opportunistic pathogens) revealed that they were all resistant to the specific molecule with diameters of inhibitions varying from 0 ± 0 to 13 ± 0 mm.

##### Activities of Aqueous Extracts of Plants on Escherichia Strains

The results show that the diameters of inhibition of the growth of species of the genus Escherichia varied from 0 ± 0 to 13.32 ± 1.2 mm (Table 5). It appears that the aqueous extract of the pulp of Alchornea cordifolia fruits had the highest activity. The antibacterial activity was greater in *E. coli* (13.32 ± 1.2 mm) than in Escherichia vulneris (11.55 ± 1.3 mm). Furthermore, the smallest inhibitory effects were induced by the extract of the leaves of Sida acuta: 8.01 ± 0.3 for *E. coli*, 8.25 ± 0.6 mm for Escherichia vulneris, and 8.31 ± 0.6 mm for Persea americana. On the other hand, the aqueous extracts of Santiria trimera, Carica papaya and Cissus leonardii showed no activity on these strains. Furthermore, the analysis of one-factor variance showed that there was a significant activation when induced by plant extracts on species of the genus Escherichia (*p* < 0.05). However, the Tukey parity test indicated no significant activity induced by extracts from the plants Geophilarenaris, Tetracera alnifolia and Anonidiummannii on the two species of the genus Escherichia.

##### Activities of Aqueous Extracts of Plants on the Klebsiella oxytoca Strain

The mean inhibition diameters of Klebsiella oxytoca growth were between 0 ± 0 and 13.68 ± 0.9 mm. The extract of the leaves of Tetracera alnifolia presented the greatest level of activity (13.68 ± 0.9 mm), followed by those of extracts of Anonidium mannii (12.09 ± 0.7), Persea Americana (11.91 ± 0.5), Geophila renaris (9.10 ± 1.3 mm) and fruit pulps of Alchorne cordifolia. However, the aqueous extracts of Santiria trimera, Carica papaya, Cissus leonardii, Sida acuta and leaves of Alchornea cordifolia showed no detected activity. In contrast, the one-factor ANOVA statistical test showed very significant inhibition diameters induced by plant extracts on Klebsiella oxytoca (*p* = 1.78 × 10^−6^). However, Tukey’s test did not show any significant level of inhibition caused by the extracts of Anonidium mannii plants and Tetracera alnifolia for Klebsiella oxytoca.

##### Activities of Aqueous Extracts of Plants on the Raoultella ornithinolytica Strain

The aqueous extracts of the leaves of Alchornea cordifolia, Santiria trimera, Carica papaya papaya and Cissus leonardii had no apparent activity in *Raoultella ornithinolytica*. On the other hand, extracts of the leaves of *Tetracera alnifolia* (11.83 ± 0.8), *Persea Americana* (11.31 ± 1.1), the fruit pulps of *Alchornea cordifolia* (10.23 ± 0.9), the leaves of *Geophila renaris* (9.87 ± 2.0) and the bark of *Santiria ebo* (9.77 ± 0.5 mm) exhibited obvious antibacterial activity. A low level of activity was recorded for Sida acuta extracts (8.36 ± 0.5 mm) and *Anonidium mannii* (8.05 ± 0.5 mm). However, the one-factor ANOVA test showed that there was a significant inhibition diameter induced by plant extracts on Raoultella ornythinolytica (*p* = 0.009). Furthermore, the extracts of the pulps of the fruits of Alchornea cordifolia, of the leaves of Tetracera alnifolia and Geophila renaris, and of seeds of Persea americana showed no statistical significanceafter the Tukey parity test (*p* > 0.05). The same was true for plant extracts from Anonidium mannii, Santiria ebo and Sida acuta (*p* > 0.05).

##### Activities of Aqueous Extracts of Plants on the Strain of Serratia odorifera

The results emphasize that the diameters of growth inhibition of Serratia odorifera vary from 0 ± 0 to 14.14 ± 0.5 mm (Table 5). Moreover, the aqueous extract of the leaves of Anonidium mannii had the highest activity (14.14 ± 0.5 mm). The appreciated inhibitory effects were recorded for the extracts of Alchornea cordifolia (7.37 ± 0.6), Santiria ebo (7.66 ± 0.6) and Geophila renaris (8.95 ± 0.0 mm). On the other hand, the aqueous extracts of Santiria trimera, Carica papaya and Cissus leonardii had no detectable activity on this strain. The one-factor ANOVA analysis of variance shows that there was a very significant difference between the activations induced by plant extracts on Serratia odorifera (*p* = 3.01 × 10^−11^). However, the Tukey test showed that there was no significant level of activity induced by the extracts of the plants of leaves Alchornea cordifolia and Geophila renaris (*p* > 0.05).

#### 2.3.2. Enteric Pathogens

Overall analysis of the antibacterial properties of the plants used in the treatment of diarrhea tested in this study against enteric pathogens indicated that, with the exception of the extract of the roots of Carica Papaya, which had no inhibitory effect, all the other plants had an apparent level of antibacterial activity on at least one of the five strains. Indeed, the extracts of the pulps of the fruits of Alchornea cordifolia, of the leaves of Santiria ebo, Anonidium mannii, Tetracera alnifolia, Sida acuta, Geophila renaris, and of the seeds of Persea americana acted on all the five enteropathogens. However, the extracts of the leaves of *Alchornea cordifolia*, *Santiria trimera* and *Cissus leonardii* only showed antibacterial activity in *Shigella sonnei*, *Shigella* sp. and *Yersinia pestis*. The aqueous extract of the leaves of Sida acuta showed the strongest level of antibacterial activity for *Shigella* sp. (16.35 ± 0.5) and *Yesrsinia pestis* (16.22 ± 0.7), and the latter was superior to those of the reference antibiotic (gentamicin).

##### Activities of Plant Extracts on Salmonella

The results reveal that the aqueous extracts of the leaves of Alchornea cordifolia, Santiria trimera and Cissus leonardii, and that of the roots of Carica papaya, did not act on any of the strains of the genus Salmonella (Table 5). In addition, our results highlighted a remarkably high level of activity, which was induced by the aqueous extract of the leaves of Anonidium manni and that activity was greater with Salmonella Paratyphi (12.79 ± 0.6 mm) when compared to that of Salmonella enterica (11.41 ± 1.1 mm). The smallest inhibitory effect (9.52 ± 0.7 mm) was induced by the extract of the pulp of the fruits of Alchornea cordifolia on Salmonella enterica (Table 5). From a statistical point of view, the analysis of variance with the ANOVA test showed that there was a significant level of inhibition of Salmonella (*p* = 0.04).

##### Antibacterial Activities of Plant Extracts on Shigella

Table 5 also shows that only the aqueous extract of the roots of Carica papaya had no level of activity when looking at bacteria of the genus Shigella. The aqueous extract of Sida acuta showed the highest level of antibacterial activity. The latter was stronger on Shigella sonnei (16.22 ± 0.7 mm) than on Shigella sp. (13.32 ± 0.9 mm). Furthermore, the lowest level of antibacterial activity (9.23 ± 1.5 mm) was recorded with the extract of the pulp of the fruits of Alchornea cordifolia on Shigella sonnei (Table 5). However, the one-factor ANOVA test showed that there was a very significant level of inhibition induced by plant extracts on Shigella (*p* = 7.65 × 10^−6^).

##### Activities of the Five Plant Extracts on Yersinia

The analysis of the antibacterial activity of the plants used in the treatment of diarrhea on the strain of Yersinia pestis revealed that, with the exception, of the aqueous extract of the roots of Carica papaya, all the other extracts had antibacterial activity on this bacteria strain. In addition, the aqueous extract of the leaves of Sida acuta showed the highest level of antibacterial activity (16.22 ± 0.7 mm). On the other hand, the lowest level of activity was recorded with the aqueous extract of Anonidium mannii (11.63 ± 0.7 mm). The one-factor analysis of variance showed that there was a significant level of inhibition induced by plant extracts in Serratia odorifera and Yersinia pestis (*p* = 9.5 × 10^−6^). Furthermore, Tukey’s parity test showed that there was no inhibition induced by plant extracts fruits and leaves of Alchornea cordifolia, Anonidium mannii and Tetracera alnifolia in Yersinia pestis (*p* > 0.05). Similarly, the Tukey test did not show the inhibition diameters induced by plant extracts of Santiria trimera, Persea americana and Cissus leonardii (*p* > 0.05).

#### 2.3.3. Comparison between the Two Types of Bacteria

The global analysis of the antibacterial activity of plant extracts on opportunistic pathogens and strict pathogens revealed that (1) the high levels of activity were recorded with strict pathogens, in particular *Yersinia pestis* (16.35 ± 0.5 mm), *Salmonella* sp. (16.22 ± 0.7 mm) and *Shigella sonnei* (14.23 ± 0.7 mm) for the extracts of Sida acuta, Anonidium mannii and Santira ebo, respectively; and (2) the aqueous extract of Carica papaya had no apparent level of activity on both types of bacteria, while the extracts of *Santiria trimera* and *Cissus leonardii* showed no level of activity, mainly for opportunistic pathogens.

## 3. Discussion

This study carried out an ethnobotanical survey to inventory the medicinal plants used to treat diarrheal infections in the Haut-Ogooué and Ogooué-Lolo provinces in Gabon. Equally, phytochemical screening was performed to show their antibacterial properties. Contrary to what has been reported in other studies, the results of the ethnobotanical survey revealed that the percentage of people (55.56%) with knowledge of at least one plant that treats diarrhea was higher than that (44.54%) of those who did not know any herbal remedy. This finding could be attributed to the ancestral practice and the great use of medicinal plants to treat diarrhea in children under 5 years old [2,9,10], coupled with the lack of appropriate hospital structures, especially in rural and semi-rural areas [11].

The decreasing order of the medicinal plant parts used by the populations of southeastern Gabon to treat diarrheal infections is as follows: bark (39.5%), leaves (34.2%), roots (7.9%), fruits (7.9%), and the rest were used by 3%, including seeds, core, twig leaf and trunk hearts. These results are different from those reported in previous studies, which designated the leaves as the most frequently used plant part for preparations of traditional medicines [12,13,14]. On the other hand, the methods of preparation in this work were mainly decoction (26.3%) and bark powder (28.9%). This observation correlates and is in line with those of many studies that indicated that decoctions were more commonly used in traditional medicines [12,13,14]. Moreover, works carried out in West and Central Africa underlined that Alchornea cordifolia, Bridelia ferruginea, Euphorbia hirta, Khaya senegalensis, Cryptolepis calophylla and Psidium guajava were mainly used in decoction by phytotherapists when treating diarrhea [15,16,17]. However, in Madagascar, Nicolas and co-workers (2012) also showed that Psidium guayava and Euphorbia hirta were used as decoctions for the treatment of diarrhea [18]. The use of a decoction makes it possible to collect high concentrations of active compounds and reduce or cancel the toxic effects of certain plants [19]. In addition, the high use of bark powders is explained by the fact that this gallic form often facilitates the administration of active principles without altering their effects [20]. Equally, preparations are mainly administered orally because it allows drugs to reach internal organs where microbial agents are accidentally or permanently located. However, the present study indicates that chewing (44.7%) followed by drinking (36.8%) are the most widely used modes of oral administration. This finding is contradictory to several studies [13,14], which have shown that drinks (decocted or macerated) are the preferential modes of administration when considering traditional medicine.

The phytochemical assessment of the 20 aqueous extracts of plants reveals that these extracts were very rich in secondary metabolites, particularly in terpenes/sterols, polyphenols, tannins, alkaloids and saponosides but also had high concentrations of anthracene compounds, cardiotonic heterosides and reducing sugars. These chemical compounds are known to have antimicrobial properties in many microorganisms [21,22,23]. Indeed, it has been shown in the literature that phenols, flavonoids and alkaloids can reduce or eliminate infectious microorganisms and have phospholipid activity [24]. Furthermore, it has been demonstrated by numerous works that the anti-diarrheal properties of medicinal plants are conferred by the presence of alkaloids, saponosides, terpenoids, flavonoids and sterols [25,26,27]. These phytomolecules are able to absorb electrolytes and water at the intestinal lumen [25], thus decreasing gastrointestinal motility [27]. The presence of these metabolites in plants used for the treatment of diarrhea by the populations in the Haut-Ogooué and Ogooué-Lolo provinces in Gabon suggests their anti-diarrheal properties and proven efficacy. In addition, the differences in phytochemical composition observed could be justified by the parts, the specific richness, the family, the photosynthetic capacity and the geographic location of each plant [13,28].

Of the 33 herbal medicines used in the treatment of diarrhea in this study, the antibacterial activity or the inhibitory power of 10 plants was evaluated on clinical isolates of infant diarrheal feces. In light of our results, it appears that the inhibitory power of the aqueous extracts of these plants on the germs tested is heterogeneous. The inhibition of the growth of bacteria could be explained by the richness and high contents of plants secondary metabolites, such as tannins, alkaloids, flavonoids, saponosides and terpene compounds. Several authors have reported that these chemical compounds have, among other things, antibacterial properties [21,23]. In addition, these antibacterial activities may be based on the mechanisms of action of phytomolecules. These have been shown to destroy the external and internal membranes of bacteria by a lipolytic action that induces a leak in the intracellular material [23,24]. Indeed, work carried out on the antibacterial activity of curcumin I has shown the destabilization of the membranes of both Gram + and Gram- bacteria [24]. Furthermore, the capacity of plant extracts in reducing bacterial growth (low activity or high activity) could party be explained by the variation in their concentrations of secondary metabolites, whose concentrations would be closely related to the harvesting locations and the intrinsic characteristics of the plants. For example, in this study, the two species of the Santiria complex (Santiria ebo and Santiria trimera), although belonging to the same genus, do not have the same qualitative phytochemical composition, and this difference is reflected in their inhibitory effect on the growth of pathogens. Additionally, ecosystem parameters play a fundamental role in plant phytochemistry and are responsible for the antimicrobial activity of plants [29]. It also depends on the part of the plant used. As a result, the aqueous extract of Alchornea cordifolia fruit pulp had antibacterial activity on all strains with a greater inhibitory power (13.32 ± 1.2 mm) on Escherichia coli, while the leaf extract had activity only on Escherichia vulneris, Serratia odorifera, Shigella sonnei, Shigella Spp. and Yersinia pestis. Similar results were found with the fruit pulps of Alchornea floribonda [30].

On the other hand, the hypothesis of the consequence of the establishment of several resistance mechanisms by bacteria is supported by the phytochemical results for the leaves of Alchornea cordifolia, Santiria trimera, Cissus leonardii, which do not reduce the growth of *Escherichia coli*, *Escherichia vulneris*, *Klebsiella oxytoca*, *Raoultella ornithinolytica*, *Serratia odorifera*, *Salmonella enterica* and *Salmonella Paratyphi*, whereas they do inhibit that of *Shigella Sonnei*, *Shigella* spp. and *Yersinia pestis*. In addition, this bacterial-growth-inhibiting power is higher in strict pathogens than in opportunistic pathogens. In fact, extracts from the leaves of Sida acuta from Santiria ebo, from Tetracera Alnifolia, from Geophila renaris, from the bark of Anonidium mannii, from seeds of Persea americana and pulps from the fruits of Alchornea cordifolia have shown the greatest inhibiting activity on *Serratia odorifera*, *Salmonella enterica*, *Salmonella Parathyphi*, *Shigella* spp., *Shigella sonnei* and *Yersinia pestis*. Moreover, all the extracts, except that of Carica papaya which does not have antibacterial activity on opportunistic pathogens, have activity on strict pathogens *Shigella* spp., *Shigella sonnei* and *Yersinia pestis*. These results suggest that opportunistic enteropathogens have developed elaborate resistance mechanisms compared to pathogens that have been declared in the literature to be the most resistant [31,32]. Indeed, the environment offers a favorable environment for horizontal exchanges of genetic material between strict pathogens, which constitute a reservoir of mobile genetic elements and non-pathogenic or opportunistic pathogens. Several studies have claimed that mobile genetic elements, such as integrons and plasmids, carry resistance and virulence genes [33,34]. Ultimately, the resistance genes that only affect pathogenic bacteria can also spread among opportunist agents. Finally, the comparison of the inhibiting activity between the reference antibiotic (gentamicin) and the aqueous extracts of plants underlined that the inhibitory capacity of plant extracts is higher (0 ± 0.0 to 16.35 ± 0.5 mm) than that of the antibiotic (0 ± 0.0 to 13.60 ± 60 mm). These results justify the use of medicinal plants by the populations of southeastern Gabon in the treatment of diarrheal diseases for both children and adults.

## 4. Materials and Methods

### 4.1. The Study Area

**Figure 1:**
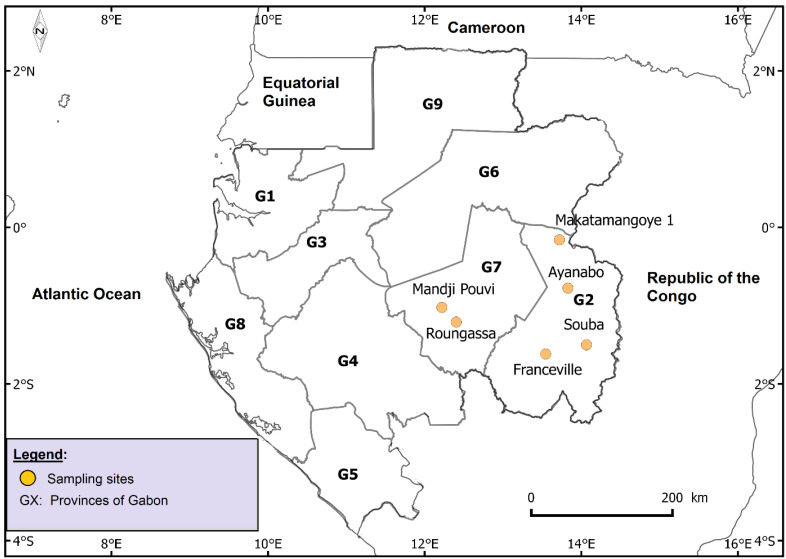
Map of Gabon showing the six localities included in the study and the nine provinces of the country. G1: Estuaire; G2: Haut-Ogooué; G3: Moyen-Ogooué; G4: Ngounié; G5: Nyanga; G6: Ogooué-Ivindo; G7: Ogooué-Lolo; G8: Ogooué-Maritime; G9: Woleu-Ntem.

The study was carried out in six localities in two provinces (Haut-Ogooué and Ogooué-Lolo provinces) in southeastern Gabon (Figure 1). Four of the six localities are found in the Haut-Ogooué province: Franceville, the provincial capital (1°37′15″ S and 13°34′58″ E), and three villages, namely Ayanabo (0°46′32” S and 13°49′49″ E), Makatamangoye I (0°09′33″ S and 13°43′14″ E) and Souba (1°30′00″ S and 14°04′00″ E). The other two localities are villages found in the Ogooué-Lolo province: Roungassa (1°01′22″ S and 12°13′13″ E) and Mandji Pouvi (1°12′36″ S and 12°24′13″ E).

Gabon is a Central African country covering a total land area of 267,667 km^2^. It is bordered to the north by the Republic of Equatorial Guinea and Cameroon, to the east and south by the Republic of Congo, and to the west by the Atlantic Ocean (Figure 1). Rainforest covers 80% of the national territory and there is great forest heterogeneity [35]. For example, the Haut-Ogooué (36.547 km^2^) and Ogooué-Lolo (25.380 km^2^) provinces have dense lowland forests, degraded forests and shrub savannas with Phyllantaceae. Southeastern Gabon is covered by three areas, namely the Ogooué Valley, the Massif du Chaillu and the Batéké Plateaux, with altitudes ranging between 350 m and 1000 m, an annual rainfall ranging between 1800 mm to 2750 mm, average temperature varying between 24 °C and 25 °C (minimum temperature of 18 °C and maximum of 35 °C) and calcareous, ferritic and sandy soils [35].

The Haut-Ogooué province includes 11 departments and has a population of 251,000 inhabitants. The Ogooué-Lolo province includes four departments and has a population of 66,000 inhabitants. Historically, the first settlers of southeastern Gabon were populations of the Babongo ethnic subgroup (pygmies), who were followed by the Nzébi, Awandji, Adouma, Obamba, Ndoumou, Bakanigui, Batéké and Bahoumbou ethnic groups (Raponda-Walker and Sillans, 1961; Deschamps, 1962). Currently, many other ethnic groups are found in this part of country.

### 4.2. Collection of Ethnopharmacology Data

#### 4.2.1. Interview and Field Survey

The first step of the study consisted in collecting ethnopharmacology data through semi-structured interviews. However, no interviews were conducted in the Souba locality. According to the legal and ethical regulations mentioned within Law N°22/2000 determining the fundamental principles of scientific research in Gabon, each participant was informed about the aims of study and only consenting participants were considered. Questions were focused on local plant names used in traditional medicine, the parts used, the method of preparation and administration and the types of diseases treated, including diarrheal conditions.

Data collection was performed from 26 October to 14 December 2017 (8 weeks). Any participant, over 18 years of age, who had knowledge or not of medicinal plants used to treat diarrhea, colic and dysentery was included in the study. Participants were randomly selected and included individuals who came across the visited areas of the homes in the five villages, since the numbers of houses were generally low (less than 100). On the other hand, in Franceville, a city with a very high number of homes and easy access to modern health facilities, the main group of participants interviewed was traditional therapists.

After the interview phase, a field study was conducted to search for the plant species cited by the interviewees. These were collected when found and identified. However, only mature fruits of Alchornea cordifolia (Euphorbiaceae) were collected in Souba. Informants usually used the vernacular names of species; however, the field survey made it possible to establish correspondence between the vernacular name and the Latin name for each plant. For plants that were difficult to access, a local guide with a good knowledge of the listed plants according to vernacular names by interviewees was hired to help the team find the plants in the field and facilitate the search. Thus, vouchers of no common species were made and kept in different herbariums: BR, BRLU, LBV, MO, P, WAG, acronyms following [36,37] and in the herbarium of the Biology Department of University of Sciences et Techniques of Masuku (USTM) in Franceville, Gabon. Most of those vouchers are available online following the link http://www.tropicos.org (accessed on 1 May 2023) of the Missouri Botanical Garden database (TROPICOS^®^).

#### 4.2.2. Selection of Plants

The selection of plants was carried out according to of four basis criteria: (1) the frequency of use of a plant by population according to the interviewees; (2) the “preferred” character of the plant as a last bulwark; (3) ease of accessibility of the plant species and samples during field collection; and (4) comparison with what had already been conducted by other authors or degree of novelty.

Preparation of aqueous plant extracts.

The collected plant material was washed and dried at an ambient temperature out of direct exposure to sunlight for two weeks. Resulting powders were transferred to sterile and dry glass jars and then stored at 4 °C until use. These powders were used to prepare all aqueous extracts.

A total of 100 g of powder was macerated in 120 mL of distilled water for approximately 18 h with magnetic stirring at room temperature. The filtrate obtained was then lyophilized (Labconco Free Zone 74200, Kansas, MO, USA) at −42 °C for 48 h. This lyophilizate was used for microbiological tests. For each plant extract tested, 100 mg of lyophilized powder was dissolved in 1 mL of a hydro-ethanolic mixture prepared from 75 mL of sterile distilled water and 25 mL of 70% ethanol.

#### 4.2.3. Phytochemical Screening

The qualitative composition of the aqueous extracts plant was carried out to test for the presence or not of large families of secondary metabolites, such as alkaloids, flavonoids, polyphenols, saponosides, sterols, terpenes, tannins and reducing sugars [38,39].

### 4.3. Microbiological Analysis

#### 4.3.1. Microbial Materials

Ten (10) bacterial strains isolated from infantile diarrheal feces collected at the Paul Moukambi Regional Hospital Center in Koula-Moutou (Ogooué-Lolo province) were used as microbial materials in this study. The strains belonged to the Enterobacteriaceae family and were divided into seven (7) genera (Escherichia, Klebsiella, Raoultella, Salmonella, Shigella, Yersinia), which included the following species: *Escherichia coli* and *vulneris*, *Klebsiella oxytoca*, *Raoultella ornithinolytica*, *Salmonella enterica* and *Paratyphie A*, *Shigella sonnei* spp. and *Yersinia pestis*). These microbial strains are now part of the collection of the Laboratory of Molecular and Cellular Biology (LAMCB) of the USTM Biology Department.

#### 4.3.2. Antimicrobial Activities

Antimicrobial activity was assessed using the well diffusion method on Mueller Hinton (MH) agar. The MH was first poured into Petri dishes and then inoculated by flooding with 2 mL of an inoculum of 2. 10^6^ CFU/mL. The plates were then dried for 15 min under a laminar flow hood and wells were then made. Fifty microliters (50 µL) of the extract from each plant was transferred to plates and the preparations incubated for 18–24 h at 37 °C. Antimicrobial activities were determined by measuring the inhibition diameters induced by the plant extracts or the reference antibiotic (gentamicin). For the latter, CASFM 2019 v.2.0 breakpoints were used to establish the profiles of the strains tested (susceptible, intermediate or resistant). Each test was performed 3 times (in triplicate).

#### 4.3.3. Statistical Analyzes

The one-factor ANOVA test was used to compare the inhibition diameters observed between the different bacteria. This test was followed by the Tukey parity test in order to compare the inhibition diameters of plants two by two on bacteria. This test was carried out using software R version 3.2.2. Excel software (2013) was used to process the database.

## 5. Conclusions

Our study highlights that a large number of medicinal plants are used for the treatment of diarrheal infections in southeastern provinces. In total, 33 species of plants belonging to 28 genera distributed in 20 families were identified. These plant species are rich in heterogeneous secondary metabolites and the derived aqueous extracts inhibit the growth of enteric isolates involved in infant diarrhea, with the exception of that of C. papaya. Based on these results, the use of these medicinal plants in the treatment of diarrheal infections by the Gabonese population seems to be justified. They constitute a real potential source of the metabolites involved in the physiological mechanisms participating in the reduction in diarrhea but also of the antimicrobial phytomolecules within the framework of the research of new bioactive molecules. It would therefore be interesting to extend the survey to the other provinces of Gabon to inventory other medicinal plants used in the treatment of diarrhea. In addition, further studies should quantify and characterize the secondary metabolites present in plant extracts to determine the parameters of inhibition, namely minimum inhibitory concentration and minimum bactericidal concentration. This effort validates the need for the development of improved traditional medicines (MTA), which should be promoted as the potency of the ethnopharmacological can maybe a way to overcome the persistent issue related to multi-resistant microorganism.

## Figures and Tables

**Table 1:** Distribution of interviewers by knowledge and gender within localities.

Provinces	Localities	Knowledgeable		Ignorant	
		Men n (%)	Women n (%)	Men n (%)	Women n (%)
Haut Ogooué	Franceville	4 (3.70)	6 (5.56)	7 (6.48)	5 (4.63)
	Ayanabo	7 (6.48)	10 (9.26)	10 (9.26)	6 (5.56)
	Makatamangoye 1	6 (5.56)	12 (11.11)	2 (1.85)	0 (0.00)
Ogooué-Lolo	Roungassa	4 (3.70)	7 (6.48)	4 (3.70)	14 (12.96)
	Mandji-Puvi	4 (3.70)	0 (0.00)	0 (0.00)	0 (0.00)
	Total	25 (23.25)	35 (32.41)	23 (21.30)	25(23.25)

**Table 2:** Medicinal plant used in treating diarrhea in 6 localities in Gabon.

Voucher Number	Family (%)	n	Scientific Name	Local Name (Dialect)	Organs	Mp	Ra	Co
Mokouapamba et al. 6	Achariaceae (2.6)	1	*Scottellia klaineana* Pierre	Lébôndo (Kota)	Bark	Po	Or	Che
na	Anacardiaceae (2.6)	9	* *Mangifera indica* L.	Omangou (Obamba), mundjiku-a-mutangani (Aduma)	Bark	Dec	Or	Dri
Ikabanga and Nzomba 870	Annonaceae (7.9)	11	*Annona senegalensis* subsp. *oulotricha* Le Thomas	Ngari (Bakanigui)	Leaves	Dec	Or	Dri
na		15	* *Annona muricata* L.	llanga-li-étangani (Ndumu)	Leaves	Dec	Or	Dri
Mokouapamba et al. 4		3	*Anonidium mannii* (Oliv.) Engl. & Diels	Imbèyi (Kota)	Bark	Dec/Po	Or	Che/Dri
Mokouapamba and Ikabanga 1	Asteraceae (10.5)	5	* *Ageratum conyzoides* (L.) L.	Pôta-mbwandi (kota); Gimbu-gya-mukunga (Nzebi)	Leaves	Ma	Or	Dri
Ikabanga 204		6	* *Bidens pilosa* L.	Matsi-ma-mangala (Punu)	Leaves	Dec	Or	Dri
na		3	* *Chromolaena odorata* (L.) R.M.King & H.Rob	Moanda-Moanda (Obamba)	Leaves	Cru	Or	Che
na		3	*Gymnanthemum amygdalinum* (Delile) Sch.Bip.	Kungu-bululu (Obamba)	Leaves	-	-	-
Ikabanga 63	Burseraceae (15.8)	6	*Aucoumea klaineana* Pierre	Ogoumi (Mbawè)	Bark	Po	Or	Che
Mokouapamba et al. 13		6	*Pachylobus buettneri* (Engl.) Guillaumin	I’nsio (Kota)	Bark	Po	Or	Che
Mokouapamba et al. 10		1	*Pachylobus camerunensis* (Onana) Byng & Christenh.	Ynralé (Kota)	Bark	Po	Or	Che
na		7	*Pachylobus edulis* G.Don	I’ranwu (kota)	Bark	Po	Or	Che
Ikabanga et al. 775		2	*Santiria ebo* (Pierre) H.J.Lam	Tombè (Aduma), Munyinga (Nzebi)	Leaves	Ma	Or	Dri
Ikabanga et al. 869		2	*Santiria trimera* (Oliv.) Aubrév.	I’koungou (Kota)	Bark	Po	Or	Che
na	Caricaceae (2.6)	8	* *Carica papaya* L.	Mulolo (Aduma, Nzebi)	Root	Dec	Or	Dri
Ikabanga and Mokea-Niaty 513	Dilleniaceae (2.6)	1	*Tetracera alnifolia* Willd.	Légagala (Nzebi	Fruit	-	Or	Che
Ikabanga and Mokouapamba 752	Euphorbiaceae (2.6)	6	*Alchornea cordifolia* Mûll. Arg.	Mbômbôsi (Kota)	Leaves, fruit shell	-	Or	Che
Ikabanga et al. 865	Fabaceae (5.3)	1	*Gilbertiodendron dewevrei* (De Wild.) J. Léonard	I’mbébé (Kota)	Bark	Po	Or	Che
na		13	* *Senna occidentalis* (L.) Link	Ngadi (Mbaouin), Mukèmu-mfumbi (Nzebi)	Leaves	Dec	Or	Dri
na	Lauraceae (2.6)	10	* *Persea americana* Mill.	Ovuca (Obamba)	Root core	Dec	Or	Che
Mokouapamba 11	Malvaceae (2.6)	5	*Sida acuta* Burm.f.	Ngonono (Obamba)	Leaves	Dec	Or	Dri
na	Musaceae (2.6)	2	* *Musa × paradisiaca* L.	Ikondo (Kota)	Heart of the trunk	Ma	Or	Dri
Mokouapamba et al. 14	Myristicaceae (2.6)	1	*Pycnanthus angolensis* (Welw.) Warb.	Etèyi (kota) Lelombe (Nzebi)	Bark,	Po	Or	Che
Ikabanga and Mokouapamba 753	Myrtaceae (2.6)	23	*Psidium guineense* Sw.	Ngoyavu (Aduma, Ndumu)	Twig Leaves, fruit	Dec/Ma	Or	Dri/che
na	Poaceae (2.6)	2	* *Oryza sativa* L.	Lerisi (Téké)	Seed	Dec	Or	Dri
Mokouapamba et al. 12	Rubiaceae (5.3)	1	*Geophila renaris* Hiern	Yndiba (Kota)	Leaves	Cru	Or	Che
Ikabanga and Mokouapamba 818		1	*Pauridiantha callicarpoides* (Hiern) Bremek	Mboka (Puvi)	Bark	Po	Or	Che
na	Rutaceae (2.6)	3	* *Citrus limon* (L.) Osbeck	Omoni (Obamba)	Fruit	-	Or	Dri
Mokouapamba and Ikabanga 7	Verbenaceae (7.9)	5	* *Lantana camara* L.	Inuhumba (Kota)	Root	Dec	Or	Dri
Mokouapamba and Ikabanga 8		3	*Lippia abyssinica* (Otto & A.Dietr.) Cufod.	Ndobwalola (kota), Muvèvèsè (Nzebi)	Leaves	Ma	An	Pu
Mokouapamba and Ikabanga 9		3	* *Lippia* cf. *alba* (Mill.) N.E. Br.	Ndobwalola (kota)	Leaves	Ma	An	Pu
Ikabanga and Alini 812	Vitaceae (2.6)	1	*Cissus leonardii* Dewit	Dyaba (Kota)	Leaves	In	-	-
?	Indetermined (13.2)	1		Ontsiami (Obamba)	Bark	Po	Or	Che
?		2		Letsaga (Obamba)	Bark	Po	Or	Che
?		1		Ebohomi (Kota)	Bark	Dec/Ma	Or	Dri
?		1		Mumbugu (Bambébé)	-	-	-	-
?		1		Mupossa (Nzebi)	Bark	Ca	Or	Che

na: no voucher collected; n: number of citation per species; *: introduced plants; ?: not found in the field; Mp: medicinal preparation (dec: decoction; ma: maceration; po: powder; cru: crushing, ca: calcine, in: infusion); Ra: route administration (or: oral; an: anal); Co: consumption (dri: drink; che: chewing; pu: purge).

**Table 3:** Percentage use of plant parts, medicinal preparations and consumption.

Selection	Name	Workforce (n)	%
Parts of plant	Bark	15	39.5
	Leaves	13	34.2
	Fruits	3	7.9
	Seeds	1	2.6
	Root	3	7.9
	Core	1	2.6
	Twig leaf	1	2.6
	Heart of the trunk	1	2.6
Preparation methods	Decoction	10	26.3
	Maceration	5	13.2
	Calcine	1	2.6
	Crushing	2	5.3
	Infusion	1	2.6
	Powder	11	28.9
	Mix	3	7.9
	Ind	-	13.2
Administration routes	Chewing	17	44.7
	Drink	14	36.8
	Purge	2	5.3
	Mixed	2	5.3
	Undefined	data	7.9

**Table 4:** Phytochemical screening of 20 plants used in the treatment of diarrhea.

Species	Parts of Plant	Chemical Components								
		Alkaloids	Anthracenes	Flavonoids	Cardiotonic Heterosides	Polyphenols	Saponosides	Reducing Sugars	Tannins	Terpenes/Sterols
*Ageratum conyzoides*	Leaves	+	−	+	+	+	−	−	+	++
*Alchornea cordifolia*	Leaves	++	+++	+	+	+++	−	++	+++	+++
	Fruit shell	+++	+++	++	+	++	+++	+++	+++	+++
*Anonidium mannii*	Bark	−	−	−	−	++	−	−	+	++
*Annona senegalensis*	Leaves	−	−	−	−	++	−	+++	+	+++
*Bidens pilosa*	Leaves	++	+	++	−	++	−	++	++	+++
*Carica papaya*	Roots	++	++	++	+++	++	++	+++	+	+++
*Cissus leonardii*	Leaves	++	+++	+	+	++	−	+	++	++
*Geophila renaris*	Leaves	−	−	−	−	+	+++	+++	−	−
*Gilbertiodendron dewevrei*	Bark	+	++	−	+	++	+++	+	+	+++
*Pachylobus buettneri*	Bark	++	++	+	+++	+++	+++	+	+++	+++
*Pachylobus camerunensis*	Bark	++	++	+	++	+++	+++	+++	+++	+++
*Pauridiantha callicarpoides*	Bark	+	+	−	++	++	+	+	+	+++
*Persea americana*	Seeds	+	+	−	++	++	+	+	−	+++
*Psidium guineense*	Leaves	+++	+++	+++	++	+++	++	+	+++	++
*Pycnanthus angolensis*	Bark	+++	++	+	++	++	+	+	−	+++
*Santiria ebo*	Bark	++	++	+	+	+++	++	++	+++	+++
*Santiria trimera*	Bark	+	+	+	+	++	−	+	+++	+++
*Scottelia klaineana*	Bark	−	+	−	++	++	−	+++	−	+++
*Sida acuta*	Leaves	+++	−	+	−	+	−	+	+	+
*Tetracera alnifolia*	Leaves	−	−	−	+	++	+	+++	+++	+++

−: negative test; +: weak precipitation; ++: average precipitation; +++: abundant precipitation.

**Table 5:** Antibacterial activity of plant extracts used in the treatment of diarrhea on bacterial strains in comparison with antibiotics.

Tested Molecules	Parts of Plant	Inhibition Diameters (mm)									
		Opportunistic Pathogens					Enteropathogens				
		*E. coli*	*E. vulneris*	*K. oxytoca*	*R. ornithinolytica*	*Se. odorifera* ^1^	*S. enterica*	*S. Paratyphi A*	*Sh. sonnei*	*Sh.* spp.	*Y. pestis*
GME (standard)		11.02 ± 0.7 ^R^	10.60 ± 0.7 ^R^	13.00 ± 0.0 ^R^	0.00 ± 0.0 ^R^	10.10 ± 1.7 ^R^	11.50 ± 0.8 ^R^	11.00 ± 0.2 ^R^	11.20 ± 0.3 ^R^	13.60 ± 1.2 ^R^	12.30 ± 0.5 ^R^
*Alchornea cordifolia*	Leaves	0.00 ± 0.0	7.49 ± 0.7	0.00 ± 0.0	0.00 ± 0.0	7.37 ± 0.6	0.00 ± 0.0	0.00 ± 0.0	7.74 ± 0.3	12.29 ± 1.2	12.42 ± 1.0
	Fruit shell	13.32 ± 1.2	11.55 ± 1.3	9.10 ± 1.3	10.23 ± 0.9	10.86 ± 0.7	9.52 ± 0.7	11.63 ± 1.6	9.23 ± 1.5	12.34 ± 1.5	11.76 ± 1.1
*Anonidium mannii*	Bark	11.27 ± 0.3	11.93 ± 0.6	12.09 ± 0.7	8.05 ± 0.5	14.14 ± 0.5	11.41 ± 1.1	12.79 ± 0.6	11.62 ± 0.7	14.43 ± 0.9	11.63 ± 0.7
*Carica papaya*	Root	0.00 ± 0.0	0.00 ± 0.0	0.00 ± 0.0	0.00 ± 0.0	0.00 ± 0.0	0.00 ± 0.0	0.00 ± 0.0	0.00 ± 0.0	0.00 ± 0.0	0.00 ± 0.0
*Cissus leonardi*	Leaves	0.00 ± 0.0	0.00 ± 0.0	0.00 ± 0.0	0.00 ± 0.0	0.00 ± 0.0	0.00 ± 0.0	0.00 ± 0.0	10.42 ± 0.5	10.13 ± 1.6	12.79 ± 1.5
*Geophila renaris*	Leaves	12.60 ± 0.4	11.81 ± 0.8	9.88 ± 0.3	9.87 ± 2.0	10.93 ± 0.3	11.05 ± 0.2	10.22 ± 0.0	12.31 ± 0.8	12.14 ± 0.2	13.49 ± 1.7
*Persea americana*	Seed	8.31 ± 0.6	12.01 ± 1.6	11.91 ± 0.5	11.31 ± 1.1	13.03 ± 0.5	11.55 ± 1.2	12.30 ± 0.6	10.84 ± 0.7	11.15 ± 0.7	13.17 ± 0.6
*Santiria ebo*	Bark	9.84 ± 0.7	10.78 ± 0.2	6.99 ± 0.0	9.77 ± 0.5	7.66 ± 0.6	10.77 ± 0.5	11.98 ± 0.5	14.23 ± 0.7	14.05 ± 1.4	15.51 ± 0.5
*Santiria trimera*	Bark	0.00 ± 0.0	0.00 ± 0.0	0.00 ± 0.0	0.00 ± 0.0	0.00 ± 0.0	0.00 ± 0.0	0.00 ± 0.0	10.91 ± 0.2	13.08 ± 0.9	13.90 ± 1.2
*Sida acuta*	Leaves	8.01 ± 0.3	8.25 ± 0.6	0.00 ± 0.0	8.36 ± 0.5	8.95 ± 0.0	10.68 ± 1.5	10.10 ± 0.6	13.32 ± 0.9	16.22 ± 0.7	16.35 ± 0.5
*Tetracera alnifolia*	Leaves	11.43 ± 1.3	11.77 ± 0.5	13.68 ± 0.9	11.83 ± 0.8	11.85 ± 0.3	11.38 ± 0.9	11.99 ± 1.1	12.29 ± 2.2	13.21 ± 1.0	11.90 ± 1.0

GME: gentamicin; ^R^: resistant *E. coli*: *Escherichia coli*; *E. vulneris*: *Escherichia vulneris*; *K. oxytoca*: *Klebsiella oxytoca*; *R. ornithinolytica*: *Raoultella ornithinolytica*; *Se. odorifera* ^1^: *Serratia odorifera*; *S. enterica*: *Salmonella enterica*; *S. paratyphi A*: *Salmonella paratyphi A*; *Sh. sonnei*: *Shigella sonnei*; *Y. pestis:* *Yersinia pestis*.

## Data Availability

Not applicable.
